# Hypoxia-induced microRNA-155 overexpression in extracellular vesicles promotes renal cell carcinoma progression by targeting *FOXO3*

**DOI:** 10.18632/aging.202706

**Published:** 2021-03-19

**Authors:** Liwei Meng, Zhaoquan Xing, Zhaoxin Guo, Yue Qiu, Zhaoxu Liu

**Affiliations:** 1 Department of Urology, Qilu Hospital of Shandong University, Jinan 250000, China; 2 School of Nursing, Shandong University, Jinan 250000, China

**Keywords:** extracellular vesicles, *FOXO3*, hypoxia, miR-155, renal cell carcinoma

## Abstract

Renal cell carcinoma (RCC) is a form of cancer arising from the renal epithelium, with high mortality rates that have reached stable levels over the past decade. The tumor microenvironment is an essential regulator of tumor progression and survival, and extracellular vesicles (EVs) are an important facet of this microenvironment. Herein, we explored the impact of hypoxia-induced miR-155 expression in EVs on *FOXO3* expression in RCC cells and their associated oncogenic activity. We found that RCC patients exhibited elevated miR-155 expression in EVs relative to healthy controls, suggesting that this miRNA may be important in the context of RCC progression. We then characterized EVs produced from RCC cell lines (Caki-1 and 786-O) under normoxic and hypoxic conditions, revealing that hypoxia-induced EVs contained higher levels of miR-155 and promoted cell proliferation. Then, we identified *FOXO3* as a miR-155 target. Lastly, hypoxia-induced EVs were found to be able to significantly inhibit *FOXO3* activation via facilitating miR-155 up-regulation. Together, these findings indicate that hypoxia can promote the upregulation of miR-155 in EVs and that this miRNA can act in RCC cells to suppress *FOXO3* expression, thereby enhancing cellular tumor progression.

## INTRODUCTION

Renal cell carcinoma (RCC) is a form of renal epithelial cancer that makes up 85% of primary renal tumors and 3% of all malignant tumors in adults [1]. It remains the fifth most frequently detected cancer type, and accounts for approximately 2.5% of cancer-associated mortality in Europe [2]. Globally, there were 403,262 new RCC diagnoses and 175,098 deaths [3], with the mortality rates associated with this tumor having risen to high levels that have stabilized in the past decade [4]. Many prior studies have explored genomic and epigenetic mechanisms responsible for controlling the development and progression of RCC and the surrounding immune response. In contrast, fewer studies have explored the microenvironment of RCC-associated tumor microenvironment, and the specific regulatory roles of extracellular vesicles (EVs) remain poorly understood.

EVs are small multivesicular bodies (30–150 nm in size) that are released from almost all mammalian cells [5, 6]. In general, higher EV production has been observed in cancer cells relative to healthy control cells, with these particles playing important roles in promoting or inhibiting oncogenesis, metastasis, and immunity [7]. These EVs can encapsulate different macromolecules and signaling intermediates including nucleic acids (mRNA, microRNAs [miRNAs], and DNA), proteins, lipids, and metabolites, allowing for the easy trafficking of these molecules between cells [8]. As miRNAs within EVs are stabilized by the surrounding lipid bilayer, they are of particular interest in the context of the oncogenic importance of these vesicles. miRNAs are small (~22 nucleotide) non-coding RNAs that are capable of suppressing the expression of target genes via binding to complementary 3’-untranslated region sequences in target mRNAs [9]. Patterns of miRNA expression have been found to be highly dysregulated in tumors [10]. In line with this finding, RCC patients have been found to exhibit distinct miRNA blood sample profiles as compared to healthy controls [11–14]. Multiple different tumors exhibit miR-155 upregulation, and as such it has been used as a diagnostic biomarker to detect certain cancers [15]. Previous work suggests that miR-155 upregulation also occurs in RCC [13], but its functional role in the development and progression of this tumor type remains uncertain, as does the question of whether this miRNA is incorporated into RCC-associated EVs at higher levels.

Owing to their rapid growth in size, many tumors exhibit varying degrees of hypoxia [16] that are closely linked to tumor progression [17]. RCC tumors often exhibit such hypoxic regions, and they have been linked to both therapeutic resistance and poorer patient survival. As such, hypoxia represents a valuable model for studies of the impact of the tumor microenvironment on RCC progression, allowing for further analysis of the roles of miRNA in EVs. The present study was therefore designed with the goals of isolating purified RCC-associated EVs, determining whether EV-encapsulated miR-155 plays a role in hypoxia-mediated induction of RCC progression, and examining the mechanisms whereby miR-155 impacts RCC.

## RESULTS

### The roles of RCC patient-derived EVs in RCC cells

**Figure 1:**
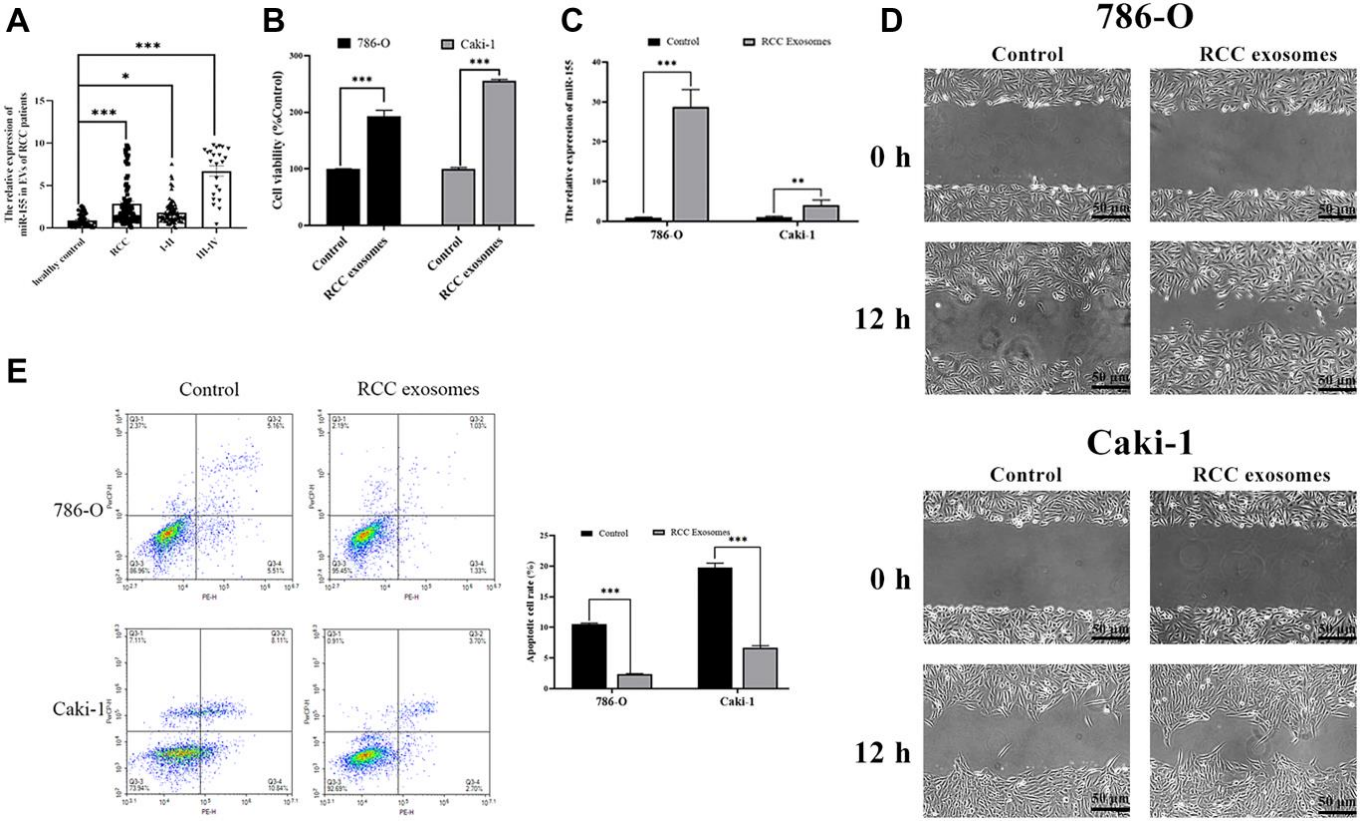
**Characterization of RCC patient EVs and their impact on RCC cells.** (**A**) RNA extracted from the RCC patient and healthy control EVs were subjected to qPCR analysis to evaluate the expression of miR-155. Levels of miR-155 increased in the RCC patient EVs. (**B**) The viability of 786-O or Caki-1 cells upon EV treatment was measured by CCK8 assay. RCC patient EVs increased cell viability. (**C**) The expression of miR-155 in 786-O or Caki-1 cells was determined by qRT-PCR after EV treatment. RCC patient EVs elevated miR-155 levels. (**D**) 786-O or Caki-1c cells were used in a wound-healing migration assay in the presence of different EVs. The migrated cells were observed under a microscope for 12 h. RCC patient EVs enhanced cell migration. (**E**) The apoptosis of 786-O or Caki-1 was measured by flow cytometry, revealing that RCC patient EVs significantly inhibited apoptosis. This experiment was conducted using three distinct biological replicates. ^*^P < 0.05, ^**^P < 0.01 and ^***^P < 0.001.

We began by measuring miR-155 expression levels in EVs isolated from RCC and healthy control patients, revealing this miRNA to be expressed at significantly higher levels in the EVs of those with RCC (Figure 1A). In addition, we found that the expression level of this miRNA rose with RCC progression. This suggests that miR-155 in EVs may play a role in controlling RCC progression. Furthermore, we assessed the impact of EVs from RCC patients. Cell viability was enhanced in the presence of EVs from RCC patients (Figure 1B). EVs from RCC patients markedly augmented the wound closure relative to EVs from healthy control patients when used to treat RCC cell lines (Figure 1D). Furthermore, EVs from RCC patients alleviated 786-O and Caki-1 cell apoptosis (Figure 1E). miR-155 levels were also elevated in RCC cell lines after treatment with EVs from RCC patients (Figure 1C). Together, these data indicated that miR-155 in EVs from RCC patients can promote RCC progression.

### Characterization of EVs derived from RCC cells

**Figure 2:**
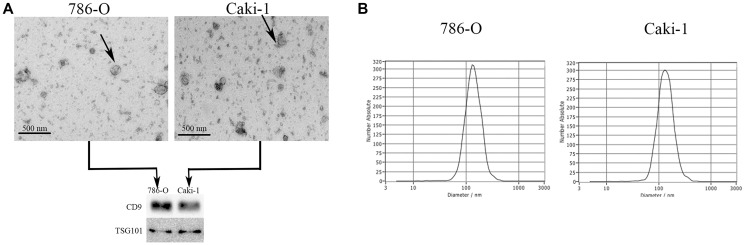
**Characterization of EVs derived from RCC cells.** (**A**) Representative EVs were analyzed via electron microscopy, and expression of the EV markers CD9 and TSG101 was assessed via Western blotting. (**B**) A NanoSight analysis was used to measure EV particle size distributions. The average particle diameters ranged 85.8–201.8 nm and 83.7–201.6 nm for EVs derived from 786-O and Caki-1 cells, respectively.

We next examined EVs produced by the 786-O (ATCC: CRL-1932) and Caki-1 (ATCC: HTB-46) human RCC cell lines. We began by assessing the morphology of these EVs via electron microscopy, and we additionally analyzed their expression of surface marker proteins including TSG101 (Tumor susceptibility protein 101) and CD9 (Cluster of differentiation 9) via Western blotting (Figure 2A). In a nanoparticle tracking analysis, we additionally measured the size and uniformity of these EVs. Overall, the average particle diameters were 144.6 nm (range: 85.8–201.8) and 143.2 nm (range: 83.7–201.6) for EVs derived from 786-O and Caki-1 cells, respectively (Figure 2B), with median concentrations of 4.2 × 10^7^ particles/mL and 3.6 × 10^7^ particles/mL, respectively.

### Hypoxia enhances EV production by RCC cells

**Figure 3:**
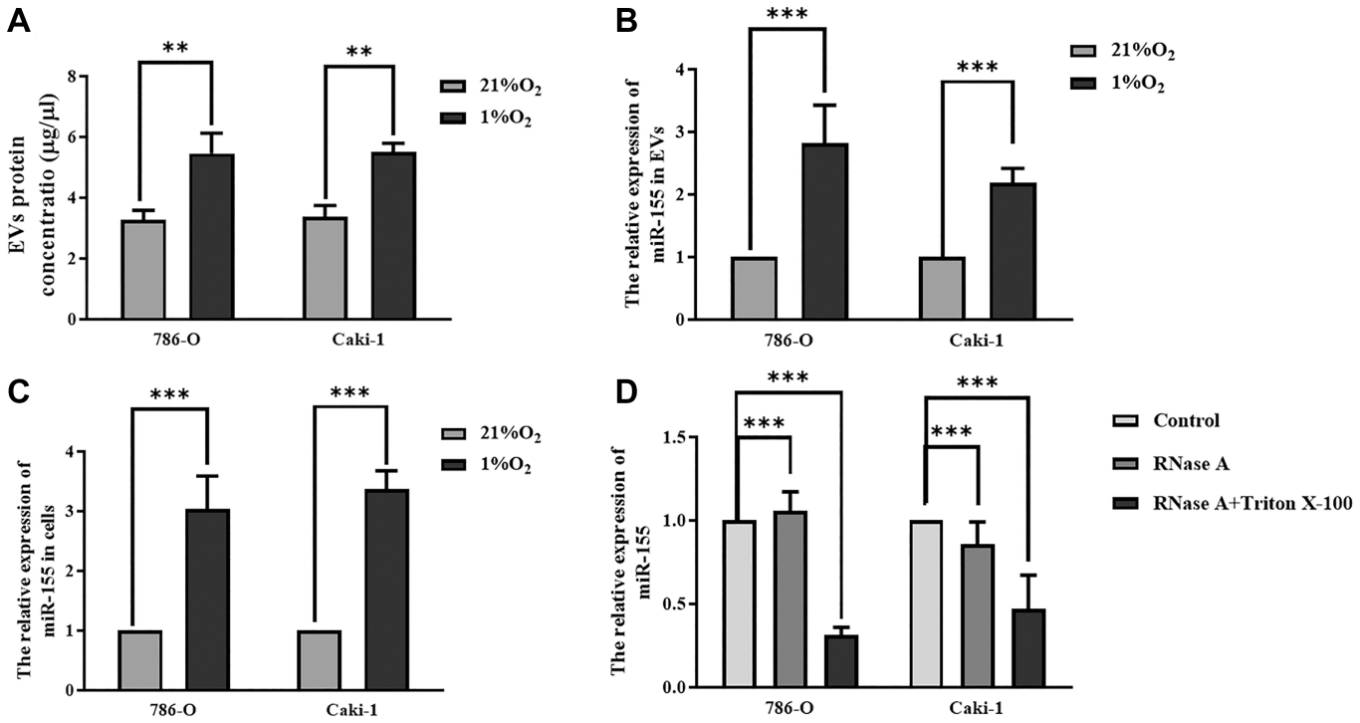
**Hypoxia impacts miR-155 expression in RCC cell-derived EVs.** (**A**) Hypoxia alters EV protein contents. (**B, C**) The impact of hypoxia on miR-155 expression in EVs (**B**) and cells (**C**). Hypoxia significantly increased protein contents in EVs. (**D**) miR-155 encapsulation within EVs was confirmed using RNase A and Triton X-100. The results confirmed that miR-155 is present primarily within EVs. This experiment was conducted using three distinct biological replicates. ^**^P < 0.01, and ^***^P < 0.001.

When we assessed protein levels within EVs derived from Caki-1 and 786-O cells, we found that hypoxia significantly increased these levels (Figure 3A), suggesting that hypoxia was sufficient to enhance EV secretion. When we measured miR-155 expression in these cells, we found that it was increased in both 786-O cells (2.03-fold) and Caki-1 cells (2.37-fold) in response to hypoxic conditions relative to normoxic conditions (Figure 3B). Similar changes in miR-155 expression were also observed in the EVs derived from these cells (Figure 3C). To further confirm that this miR-155 was encapsulated in EVs derived from these RCC cells, we treated cellular supernatants with either RNase A and/or Triton X-100. While RNase A alone failed to reduce miR-155 expression levels in these supernatants, dual RNase A and Triton X-100 treatment was sufficient to significantly reduce miR-155 expression (Figure 3D), thus confirming that this miRNA is present primarily within EVs. Together, these findings thus strongly suggested that hypoxia can promote increased miR-155 expression in RCC cells and in EVs derived therefrom.

### Hypoxia-induced EVs drive RCC progression

**Figure 4:**
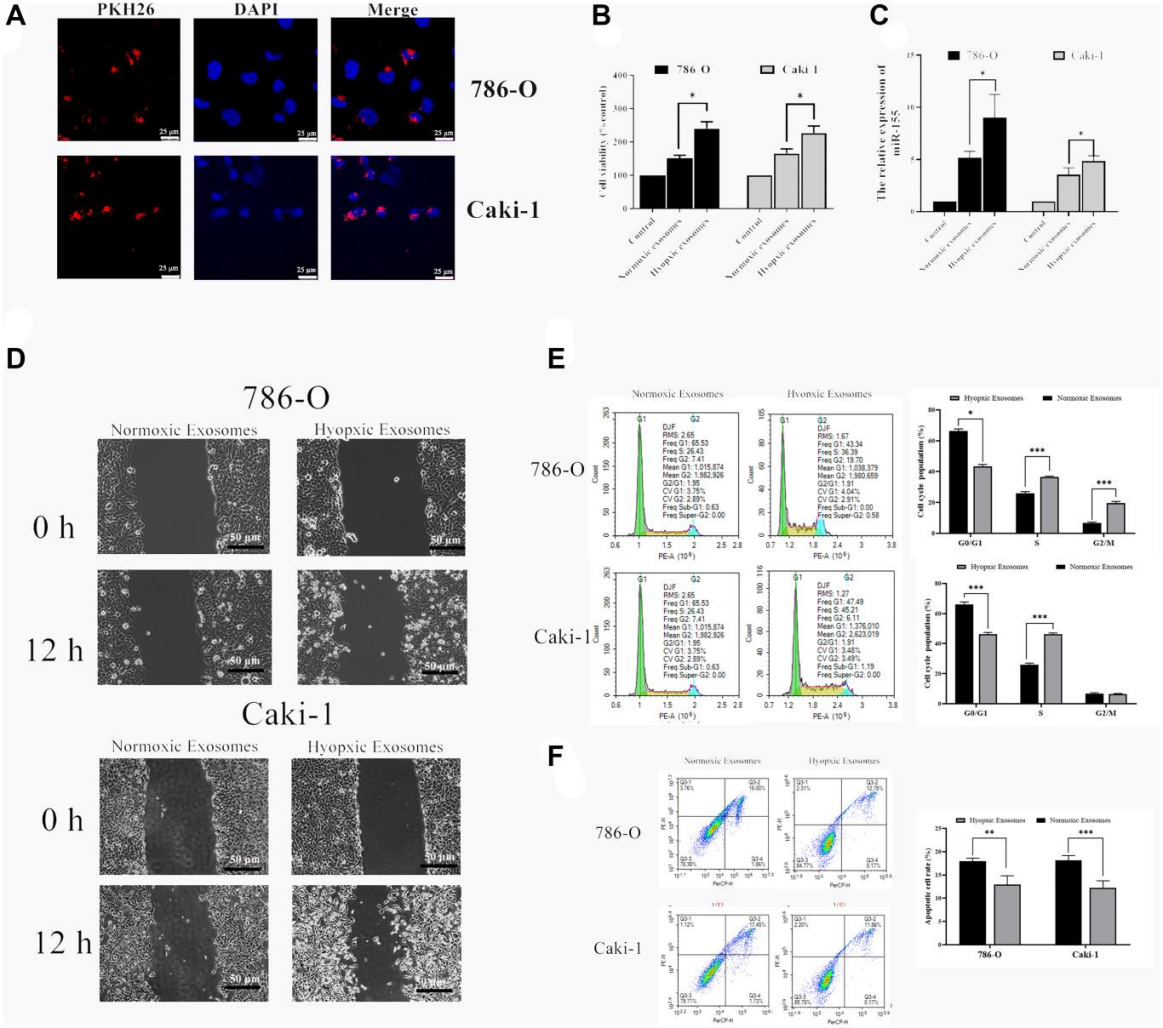
**Hypoxia-induced RCC cell-derived EVs alter the viability of 786-O and Caki-1 cells.** (**A**) EV uptake by 786-O and Caki-1 cells after 12 h was detected using PKH26-labeled EVs (red) and DAPI (blue) for nuclear localization. (**B**) The impact of hypoxia- and normoxia-derived EVs on the viability of RCC cells. The results indicated that hypoxia-induced EVs to significantly bolster the viability of treated RCC cells. (**C**) The impact of hypoxia- and normoxia-derived EVs on miR-155 levels. (**D**) Representative micrographs of the wound healing assay at 0 h and 12 h. Hypoxia-associated EVs derived from both of these cell lines were able to promote enhanced RCC cell migration. (**E**) Cell-cycle distribution was assessed by flow cytometry. Hypoxia-associated EV treatment was associated with an increased number of cells in S phase. (**F**) Apoptosis was assessed by flow cytometry. Hypoxia-associated EV treatment inhibited the cell apoptosis. This experiment was conducted using three distinct biological replicates. ^*^P < 0.05, ^**^P < 0.01, and ^***^P < 0.001.

We next explored the functional impact of hypoxia-induced RCC cell-derived EVs on the viability of these cells. To that end, we collected EVs from RCC cells grown under hypoxic conditions as above, labeled them with the red fluorescent PKH26 dye, and then added them to 786-O and Caki-1 cells for 72 h. After this time, EV uptake and nuclear localization in these RCC cells were evident (Figure 4A). We then used a CCK-8 assay to assess the viability of these cells, revealing hypoxia-induced EVs to significantly bolster the viability of treated RCC cells (Figure 4B). The migratory capabilities of these cells were then assessed in a wound healing assay. Hypoxia-associated EVs derived from both of these cell lines were able to promote enhanced RCC cell migration relative to normoxia-associated EVs (Figure 4D). The impact of these EVs on cell cycle progression and apoptosis was also assessed by flow cytometry, revealing that hypoxia-associated EV treatment was associated with an increased number of cells in S phase, consistent with enhanced cell proliferation (Figure 4D). Furthermore, hypoxia-induced EVs alleviated 786-O and Caki-1 cell apoptosis (Figure 4E). In addition, we found that miR-155 expression levels in 786-O and Caki-1 cells were 74.8% and 36.9% higher, respectively, when these cells were treated with hypoxia-derived EVs relative to when they were treated with normoxia-derived EVs (Figure 4C). Together, these results therefore suggest that hypoxia can drive RCC progression through a mechanism that may be associated with miR-155 upregulation.

### *EV-derived miR-155* enhances cellular viability

**Figure 5:**
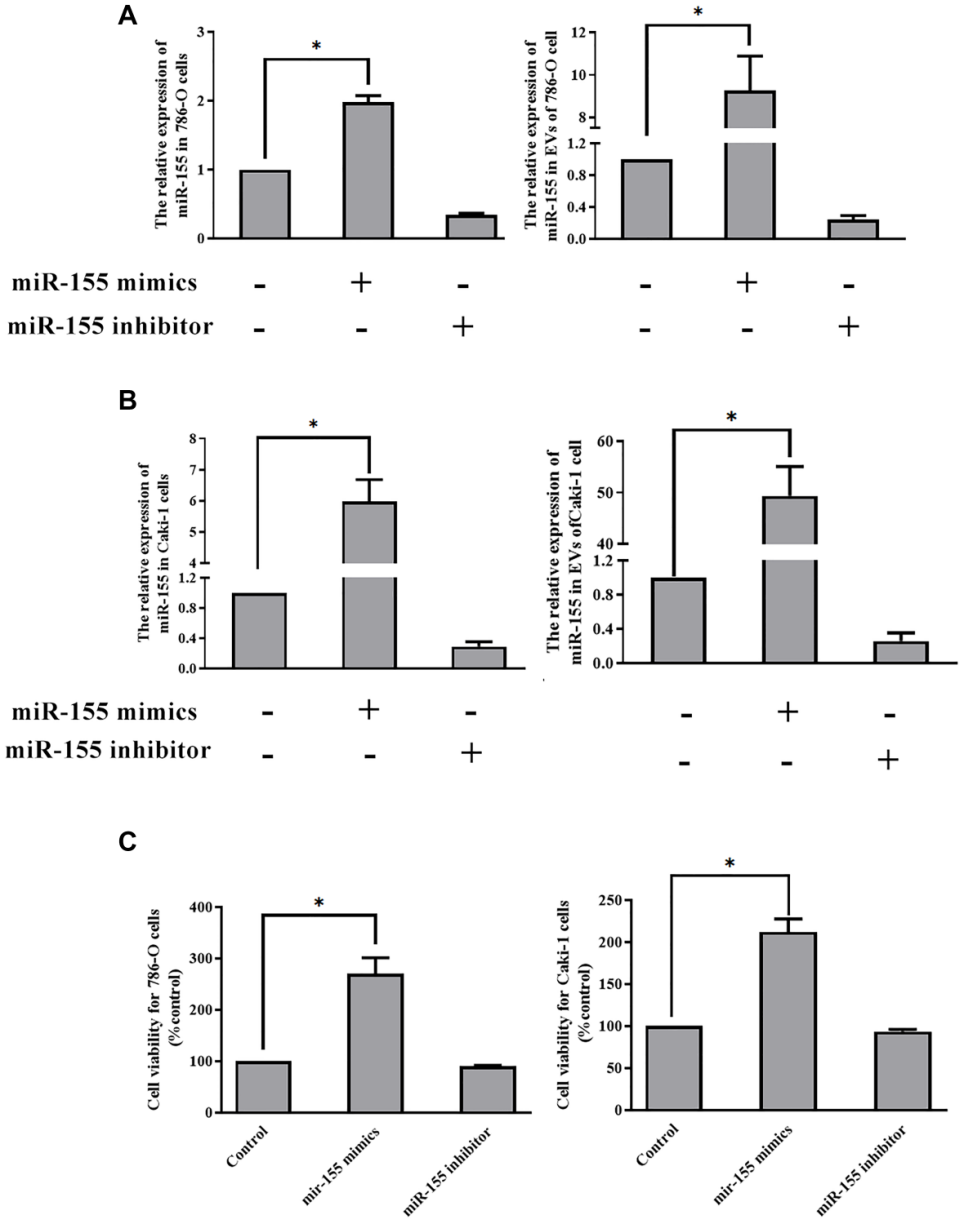
**EV-derived miR-155 alters 786-O and Caki-1 cell proliferation.** (**A, B**) Confirmation of miR-155 mimic and inhibitor transfection in 786-O and Caki-1 Cells. (**C**) The impact of miR-155 on 786-O and Caki-1 cell viability was assessed using EVs from cells prepared in A and B. EV-derived miR-155 enhances cellular viability. This experiment was conducted using three distinct biological replicates. ^*^P < 0.05.

Thus far our findings have shown that hypoxia can promote EV secretion from RCC cells and that it can induce the up-regulation of miR-155 within both these cells and the EVs derived therefrom. Furthermore, we have found that hypoxia-induced EVs can enhance RCC cellular viability. However, we have not yet demonstrated that EV-encapsulated miR-155 is responsible for this enhancement of viability. To that end, we transfected cells with miR-155 mimics or inhibitors prior to culturing them under hypoxic conditions. We confirmed that miR-155 mimics and inhibitors were able to significantly alter miR-155 expression in the expected directions in both 786-O and Caki-1 cells (Figure 5A–5B). We then collected the EVs derived from these cells and used them to treat separate 786-O and Caki-1 cultures. When we assessed the viability of these cells, we found that EVs derived from miR-155 mimic-transfected cells were able to more effectively enhance cell viability, whereas the opposite was observed when cells were treated with EVs derived from miR-155 inhibitor-transfected cells (Figure 5C).

### EV-derived miR-targets *FOXO3* in RCC cells

**Figure 6:**
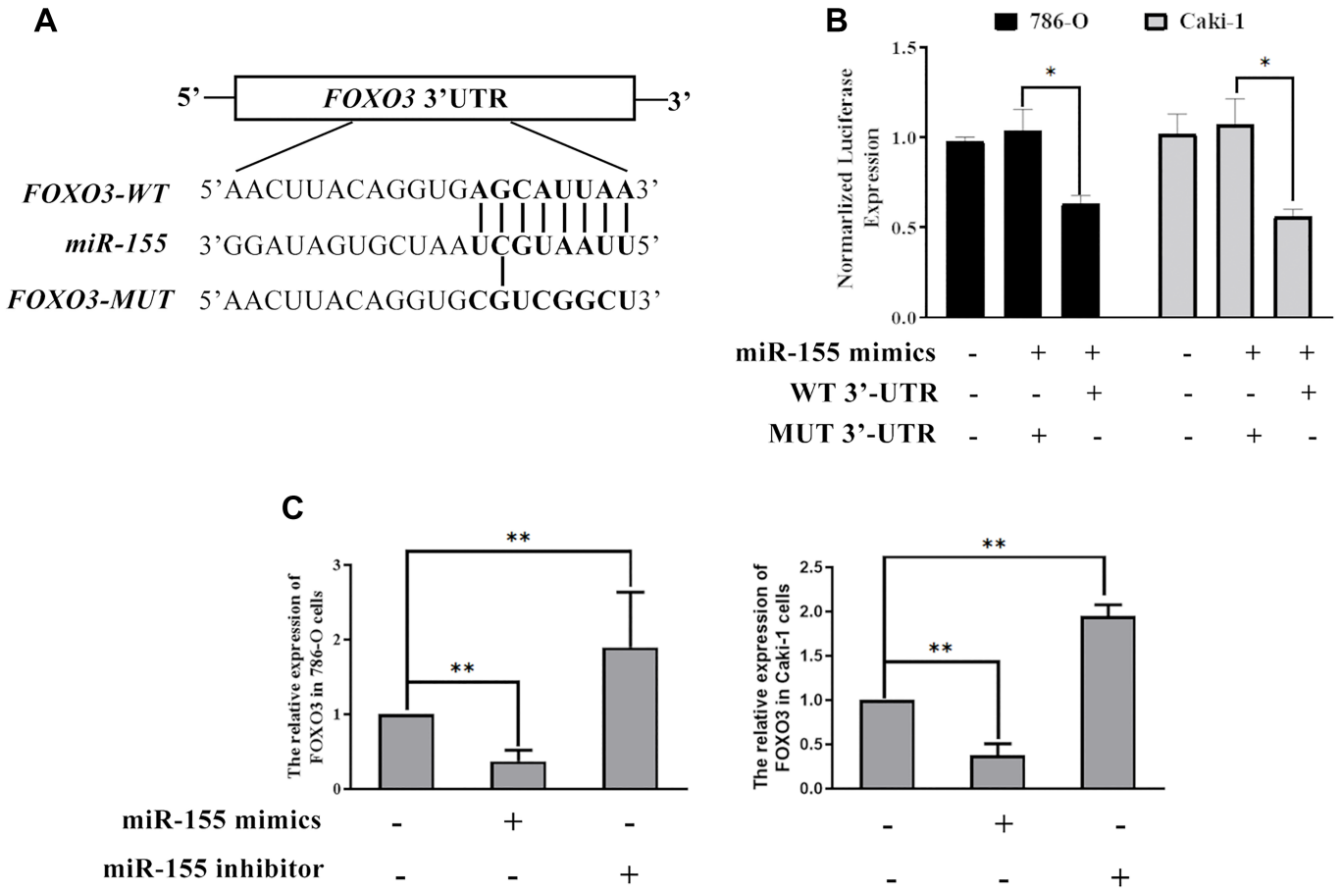
**miR-155 directly targets FOXO3.** (**A**) Predicted miR-155 binding sites in the FOXO3 3’-UTR were identified using predictive algorithms. (**B**) The activity of luciferase reporters containing WT or mutant versions of the predicted miR-155 binding site from FOXO3 was measured. (**C**) The impact of miR-155 on FOXO3 expression was quantified. This experiment was conducted using three distinct biological replicates. ^*^P < 0.05, and ^**^P < 0.01.

Next, we sought to explore the mechanistic basis whereby miR-155 modulates RCC cell proliferation. To that end, we conducted a predictive bioinformatics analysis to identify potential miR-155 target genes. This led us to identify *FOXO3* as one potential target gene of interest, as it is downregulated in RCC contains a predicted 8 bp miR-155 binding site in its 3’-UTR (Figure 6A). Based on this target sequence, we then constructed luciferase reporter constructs containing either a WT or mutant version of this binding site and then used them in a luciferase reporter assay (Figure 6A). We found that miR-155 mimics were able to significantly reduce WT but not mutant *FOXO3* 3’-UTR reporter activity in both 786-O and Caki-1 cells (Figure 6B). In addition, we confirmed that miR-155 mimic transfection was associated with a significant reduction in *FOXO3* expression, whereas the opposite was observed in response to miR-155 inhibitor transfection (Figure 6C). These findings thus confirmed that miR-155 is able to directly suppress *FOXO3* expression in RCC cells.

### *FOXO3* inhibits RCC progression

**Figure 7:**
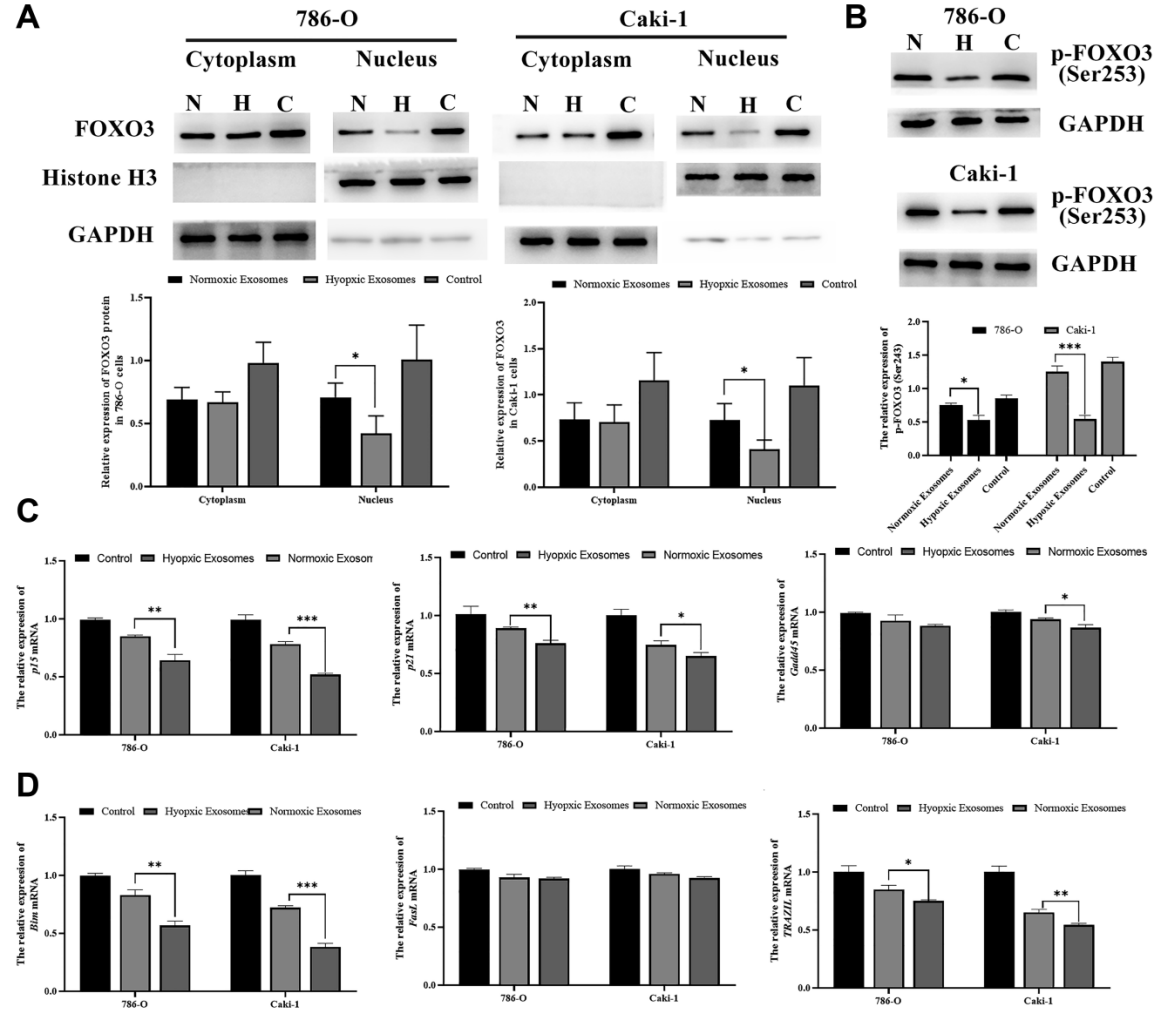
**Hypoxia-induced RCC cell-derived EVs promote RCC progression by suppressing FOXO3.** (**A**) FOXO3 localization was assessed by Western blotting, and data were quantified. The results revealed that hypoxia-induced EVs were able to significantly reduce nuclear and cytoplasmic FOXO3 expression. (**B**) FOXO3 phosphorylation was assessed by Western blotting, and data were quantified. The results indicated that hypoxia-induced EVs were able to significantly reduce the FOXO3phosphorylation levels. (**C–D**) The expression of proliferation-(**C**) and apoptosis(**D**)-related genes regulated by FOXO3. ^*^P < 0.05, ^**^P < 0.01, and ^***^ *P* < 0.001.

Lastly, we sought to determine the functional impact of altered *FOXO3* expression on RCC progression after hypoxia- and normoxia-induced EV treatment. We found that hypoxia-induced EVs were able to significantly reduce nuclear and cytoplasmic *FOXO3* expression (Figure 7A). Furthermore, hypoxia-induced EVs can also decrease the phosphorylation levels of *FOXO3* to inhibit its activation (Figure 7B). In addition, *FOXO3* target genes, including both proliferation-related genes (p15, p21, and Gadd45) and apoptosis-related genes (Bim and TRAIL) were further downregulated by hypoxia-induced EV treatment (Figure 7C, 7D).

## DISCUSSION

The existence of transformed tumor cells alone is insufficient to drive the progression of cancer [18]. Instead, microenvironmental signals including inflammatory factors and other matrix-derived factors can regulate tumor survival and progression [19, 20]. In RCC, this tumor microenvironment is a key determinant of the degree of tumor malignancy, regulating metastasis and associated tumor cell behaviors such as invasion and proliferation [21]. Hypoxia is one of the most common microenvironmental conditions observed in the context of tumor progression [18], leading to extensive changes in intratumoral gene expression and promoting immunosuppression, drug resistance, and angiogenesis [22]. Many previous studies have examined the impact of such hypoxic conditions on tumor cells, but how intratumoral hypoxia can induce malignant progression even in normoxic cells remains to be fully understood. EVs derived from tumor cells are known to be key regulators of tumor progression [23]. As such, in the present analyses we sought to explore the importance of hypoxia-derived EVs in the regulation of RCC progression.

In this study, we isolated EVs from the Caki-1 and 786-O RCC cell lines under normoxic and hypoxic conditions, revealing that hypoxia increased EV contents in line with prior studies of hepatocellular carcinoma [24] and breast cancer [25]. We additionally determined that these hypoxia-induced EVs were sufficient to enhance the proliferation of RCC cells cultured under normoxic conditions (Figure 3B–3C). EVs contain large quantities of proteins, miRNAs, and mRNAs [8]. The miRNAs present within these EVs may therefore influence the proliferation of cells into which they are internalized. A number of different miRNAs have been found to be overexpressed in RCC, including miR-155, the upregulation of which has been closely associated with hypoxia. Previous studies have shown that miR-155 plays oncogenic roles in colon cancer [26], colorectal cancer [27], gastric cancer [28], and osteosarcoma [29]. As such, we hypothesized that hypoxia may be able to induce miR-155 upregulation in RCC cells and incorporation into EVs derived from these cells, thereby achieving the observed EV-induced RCC cell proliferation effect. We were able to confirm this hypothesis using miR-155 mimics and inhibition (Figure 4). We then additionally identified *FOXO3* as a miR-155 target gene.

*FOXO3* is a forkhead box family transcription factor that is thought to regulate the expression of a number of different genes associated with cell death [30]. In the present study, we demonstrated a direct interaction between miR-155 and a target sequence in the *FOXO3* 3’-UTR, suggesting that this miRNA is able to directly target and suppress *FOXO3* expression in 786-O and Caki-1 cells. We also found that miR-155 inhibitor transfection was sufficient to enhance *FOXO3* expression levels, further confirming that *FOXO3* is suppressed by miR-155. When we overexpressed *FOXO3* in 786-O and Caki-1 cells we found that this reduced their viability, suggesting that this gene can interfere with RCC cell survival, whereas knockdown of *FOXO3* enhanced the survival of these cells.

**Figure 8:**
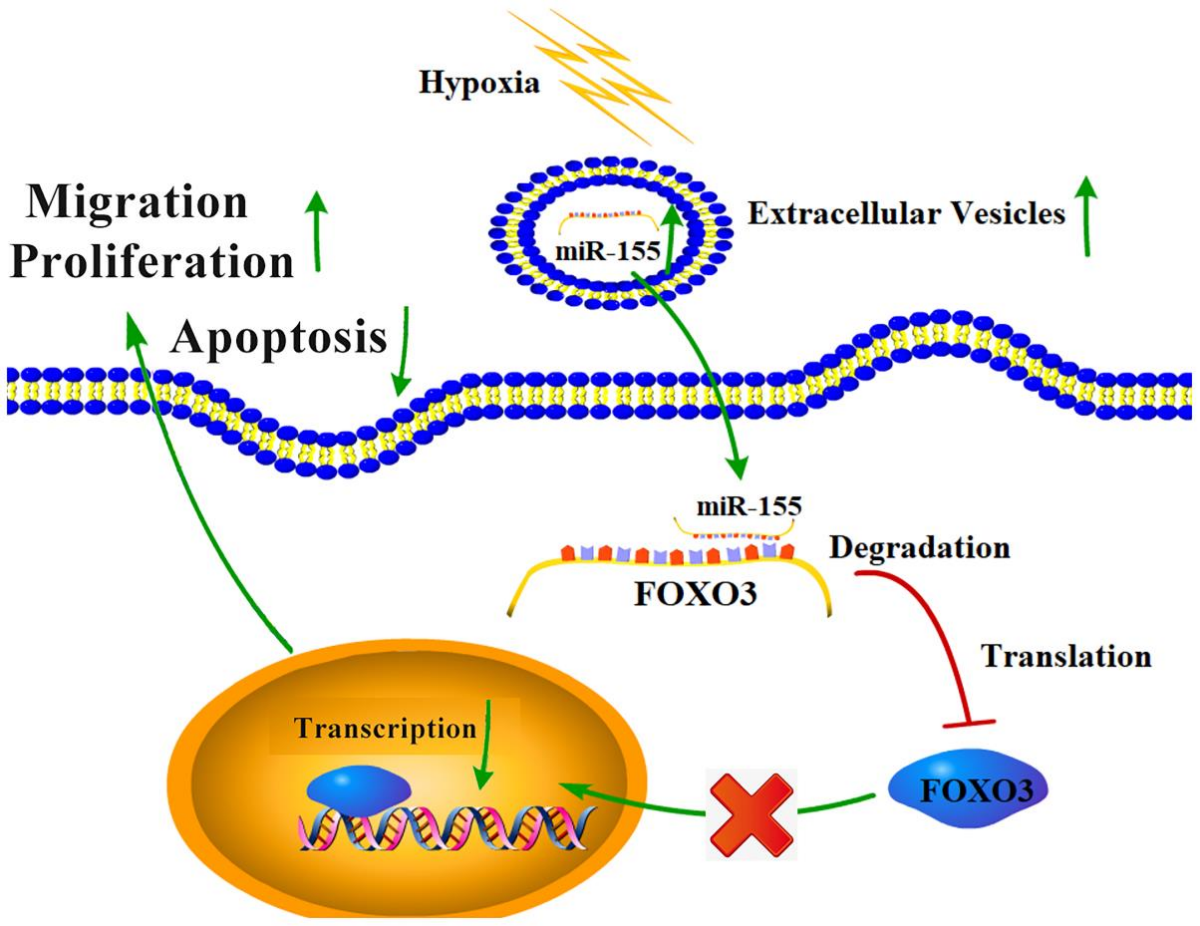
**The mechanistic basis for hypoxia-induced RCC progression.**

Together, our results highlight a hypoxia-mediated mechanism of RCC progression (Figure 8). Hypoxic conditions can promote the upregulation of miR-155 in RCC cells and in EVs. This miRNA is then able to target *FOXO3* expression in proximal or distal RCC cells, inhibiting apoptosis and promoting their migration and proliferation. These results offer new insights into the mechanistic basis of RCC, and also highlight potential therapeutic avenues for the treatment of this disease.

## MATERIALS AND METHODS

### Ethics statement

The Institutional Ethics Committee of Qilu Hospital (Shandong, P.R. China) approved all experiments conducted and patient collection described herein (approval NO. KYLL-2019-258), which were performed in line with the Helsinki declaration.

### Patients

In total, we enrolled 132 RCC patients that had been newly diagnosed at Qilu Hospital between December 2017 and August 2018. For details pertaining to clinical and pathological findings in these patients, see Supplementary Table 1. As a control, we additionally collected blood samples from 50 cancer-free healthy volunteers during this same period.

### Cell culture and hypoxia

The 786-O and Caki-1 human RCC lines were from Procell Life Science & Technology Co (Shanghai, P.R. China) and Zhongqiao Cell Research Co (Shanghai, P.R. China), respectively. Both cell lines were cultured in humidified 5% CO_2_ incubators, with Caki-1 and 786-O cells being grown in Mccoy's 5a medium and RPMI-1640 (both from Gibco), respectively, both of which were supplemented with 10% FBS. Cells were cultured under either normoxic (20% O_2_) or hypoxic (1% O_2_) conditions for appropriate experiments, with N_2_ being used to balance gas levels in a three-gas incubator (Binder).

### Isolation and authentication of EVs

The buffer XBP was combined with 250 μL serum or 100 mL of cell culture media which were filtered by 0.8 μm syringe filters, after which EVs were isolated according to provided directions (Qiagen, Germany). These EVs were then validated and analyzed via electron microscopy, Nanoparticle Tracking Analysis (NTA), and Western blotting. For transmission electron microscopy (TEM), 20 μL of an EV suspension was fixed on 100-mesh carbon-coated, formvar-coated nickel grids treated with poly-L-lysine for 30 minutes. After washing 3 times with PBS, samples were incubated with 1% glutaraldehyde for 5 min, and then were negatively stained using the aqueous 4% uranyl acetate for 5 min. The excess stain was then blotted off and the sample was air dried. Samples were then observed in a JEOL-1200EX TEM. An NTA system (ZetaView Particle Metrix, Germany) was used to assess particle size and concentrations. EV surface markers including CD9 and TSG101 were assessed via Western blotting.

### EV internalization analysis

We utilized the PKH26 dye (Sigma Aldrich, USA) to label collected EVs, which were then passed through a 10-kDa filter (Microcon YM-100, Millipore, USA) and washed thrice with PBS. These labeled EVs were then combined overnight with 786-O and Caki-1 cells in serum-free medium at 37°C. Cells were then fixed using 4% paraformaldehyde, stained using 4', 6-diamidino-2-phenylindole (DAPI; Sigma), and analyzed with an Eclipse Ti-S fluorescence microscope (Nikon, Japan).

### Transfection and treatment

For sequence details specific to this study, see Table 1. For transfection reactions, cells were plated in 96-well plates for 24 h. Lipofectamine 2000 (Invitrogen, CA, USA) was used to transfect cells with miR-155 mimics and inhibitors (GenePharma, Shanghai, China) based on provided directions. At 48 h post-transfection, we then collected these cells and purified them according to the manufacturer's instructions. The EVs collected from serum or cell culture media were combined with 786-O and Caki-1 cells in serum-free medium at 37°C for 48 h and then collected and purified according to the manufacturer's instructions.

**Table 1:** The primers and RNA sequences using in the study.

**Name**	**Sequence**	**Comments**
**FOXO3-F**	5'-CAGCCGAGGAAATGTTCGTC-3'	**qRT-PCR**
**FOXO3-R**	5'-AGAGTGAGCCGTTTGTCCG-3'	
**GAPDH-F**	5'-CAGGAGGCATTGCTGATGAT-3'	**qRT-PCR**
**GAPDH-R**	5'-GAAGGCTGGGGCTCATTT-3'	
**miR-155 mimics**	5'-UUAAUGCUAAUCGUGAUAGGGGUUCCCCUAUCACGAUUAGCAUUAAUU-3'	**mimics**
**miR-155 inhibition**	5'-AACCCCUAUCACGAUUAGCAUUAA-3'	**inhibition**
**P15-F**	5'-GGGACTAGTGGAGAAGGTGC-3'	**qRT-PCR**
**P15-R**	5'-CCATCATCATGACCTGGATCG-3'	
**p21-F**	5'-TGTCCGTCAGAACCCATGC-3'	**qRT-PCR**
**p21-R**	5'-AAAGTCGAAGTTCCATCGCTC-3'	
**Gadd45-F**	5'-GAGAGCAGAAGACCGAAAGGA-3'	**qRT-PCR**
**Gadd45-R**	5'-CACAACACCACGTTATCGGG-3'	
**Bim-F**	5'-TAAGTTCTGAGTGTGACCGAGA-3'	**qRT-PCR**
**Bim-R**	5'-GCTCTGTCTGTAGGGAGGTAGG-3'	
**FasL-F**	5'-CTCCGAGAGTCTACCAGCCA-3'	**qRT-PCR**
**FasL-R**	5'-TGGACTTGCCTGTTAAATGGG-3'	
**TRAZIL-F**	5'-TGCGTGCTGATCGTGATCTTC-3'	**qRT-PCR**
**TRAZIL-R**	5'-GCTCGTTGGTAAAGTACACGTA3'	

### Cell viability assay

In order to quantify tumor cell numbers, we utilized a Cell Counting Kit-8 kit (CCK-8; Dojindo). Briefly, we plated 5,000 786-O or Caki-1 cells per well of a 96-well plate and treated these cells as experimentally appropriate for 24 h, after which CCK-8 solution was added to each well and plates were incubated for 2 additional hours at 37°C. A microplate reader was then used to quantify absorbance at 450 nm in each well, with viability being calculated as follows: (treated−blank)/(untreated control−blank)^*^100%.

### Wound-healing assay

Cells were seeded in a 12-well plate and cultured overnight in medium containing 10% FBS. Once cells were 90–100% confluent, a scratch wound was generated in the center of each well, and serum-free media was added. Cells were then imaged at 0 and 12 h of culture following wounding using an inverted microscope, and cell migration was quantified using ImageJ.

### Flow cytometry

786-O and Caki-1 cells were seeded in 6 cm dishes and cultured in serum-free medium for 24 h before being treated with EVs for 24 h. Next, cells were harvested and fixed in 70% ethanol at 4°C for 2 h, and they were then incubated with RNase and the DNA-interacting dye propidium iodide (PI) for 30 min at room temperature. Cell cycle analyses were performed using a Guava easy Cyte HT flow cytometer (Merk, Germany).

For apoptosis analyses, 786-O and Caki-1 cells were seeded in 6-well plates and treated with EVs for 24 h. The cells were then collected, washed with PBS at 4°C, and resuspended in the 1 × binding buffer containing Annexin V-FITC and PI. After incubation for 15 min at room temperature, apoptosis was analyzed with a Guava easy Cyte HT flow cytometer (Merk, Germany). Cells in both the early and late stages of apoptosis were detected.

### Western blotting

In total, we isolated 30 μg of protein from each sample and separated this protein via 8% SDS-PAGE, after which it was transferred onto PVDF membranes (Millipore). After being blocked for 2 h, these membranes were then probed using mouse anti-CD9 or mouse anti-TSG101 (both 1:1,000, Santa Cruz Biotechnology). Blots were then probed with secondary HRP-conjugated anti-mouse IgG (1:5,000). A densitometer (GS-700; Bio-Rad Laboratories) was used to visualize protein bands in the resultant stained blots, with the Quantity One 4.4.0 software being used for quantification purposes.

### qRT-PCR

TRIzol (Invitrogen) was used to extract RNA from samples based on provided directions, after which a Nanodrop 2000 (Thermo Fisher Scientific, MA, USA) instrument was used to quantify RNA levels. A miRNA qRT-PCR Kit (Invitrogen) was used to quantify miR-155 expression levels, with U6 being used for normalization thereof. For measurement of mRNA expression levels, we utilized a TaqMan High-Capacity cDNA Reverse Transcription kit (Applied Biosystems), and β-actin was used to normalize target gene mRNA levels.

### Luciferase reporter assay

Putative miR-155 target genes were predicted using three predictive algorithms (miRTarBase, miRDB, and TargetScanHuman). To validate the interaction between this miRNA and *FOXO3*, we inserted WT or mutant *FOXO3* 3’-UTR binding sites into pmirGLO reporter vectors (Promega, WI, USA). These constructs were then co-transfected into cells along with miR-155 mimics using Lipofectamine 2000 (Invitrogen). After 48 h, we then measured luciferase activity with a dual-luciferase reporter assay system (Promega), with Renilla luciferase activity being used for normalization purposes.

### Statistical analysis

Data are means ± standard error, and all experiments were repeated three or more times. Data were compared via Student’s *t*-tests or one-way ANOVAs as appropriate, with statistical significance indicated as follows: ^*^p < 0.05, ^**^p < 0.01, and ^***^p < 0.001.

## Supplementary Material

Supplementary Table 1
